# Combined short-axis out-of-plane and long-axis in-plane approach versus long-axis in-plane approach for ultrasound-guided central venous catheterization in infants and small children: A randomized controlled trial

**DOI:** 10.1371/journal.pone.0275453

**Published:** 2022-09-30

**Authors:** Jun Takeshita, Yasufumi Nakajima, Kazuya Tachibana, Hirofumi Hamaba, Tomonori Yamashita, Nobuaki Shime

**Affiliations:** 1 Department of Anesthesiology, Osaka Prefectural Hospital Organization, Osaka Women’s and Children’s Hospital, Osaka, Japan; 2 Department of Anesthesiology, Kansai Medical University Hospital, Osaka, Japan; 3 Outcomes Research Consortium, Cleveland, OH, United States of America; 4 Department of Emergency and Critical Care Medicine, Institute of Biomedical & Health Sciences, Hiroshima University, Hiroshima, Japan; Cleveland Clinic, UNITED STATES

## Abstract

The ultrasound-guided long-axis in-plane approach for central venous catheterization in infants and small children can prevent posterior wall penetration. The combined short-axis out-of-plane and long-axis in-plane approach reportedly prevents such penetration in adults. To test the hypothesis of non-inferiority of the combined approach to the long-axis in-plane approach, we compared the two approaches in infants and small children. Patients were randomized based on whether they underwent ultrasound-guided internal jugular vein catheterization using the combined or long-axis in-plane approach. Posterior wall penetration rates, first-attempt success rates, overall success rates within 20 min; scanning, puncture, and procedure durations; and number of attempts were compared between the groups. In the combined and long-axis in-plane groups (*n* = 55 per group), the posterior wall penetration rates were 5.5% (3/55) and 3.6% (2/55) (*P* = 0.65), the first-attempt success rates were 94.5% (52/55) and 92.7% (51/55) (*P* = 0.70), and the overall success rates within 20 min were 100% (55/55) and 98.2% (54/55) (*P* = 0.32), respectively. In the combined and long-axis in-plane groups, the median (interquartile range) scanning durations were 21 (16.5–34.8) s and 47 (29.3–65) s (*P*<0.0001), the puncture durations were 114 (83–170) s and 74 (52.3–117.3) s (*P* = 0.0002), and the procedure durations were 141 (99–97.8) s and 118 (88.5–195.5) s (*P* = 0.14), respectively. The median number of attempts was 1 (interquartile range: 1–1, range: 1–3) in both groups (*P* = 0.72). Similar to the long-axis in-plane approach, the combined approach for internal jugular vein catheterization prevented posterior wall penetration in infants and small children.

**Trial registration:** This trial was registered before patient enrollment in the University Hospital Medical Information Network Clinical Trials Registry, registration number UMIN000039387 (https://upload.umin.ac.jp/cgi-bin/ctr/ctr_view_reg.cgi?recptno=R000044907).

## Introduction

The ultrasound-guided short-axis out-of-plane approach has been reported to have high success and low complication rates [1–6], and is commonly used for central venous catheterization in pediatric patients. In this approach, operators can align the site of the needle puncture with the center of the vessel with relative ease [7]. However, the position of the needle tip is occasionally missed [8], resulting in posterior wall penetration in 39.6–51% of pediatric patients [9, 10]. Preventing posterior wall penetration of the internal jugular vein is important to avoid serious complications such as pneumothorax, common carotid artery puncture, and vertebral artery puncture [11–14].

The ultrasound-guided long-axis in-plane approach for central venous catheterization can reduce posterior wall penetration compared to the short-axis out-of-plane approach in a phantom model [15], as well as in infants and small children [10]. However, this approach is not widely used in pediatric patients. Accurate visualization of the target vein and the entire needle is challenging in the long-axis view [16].

Another approach that can be utilized is the combined short-axis out-of-plane and long-axis in-plane approach [17–20]. In this approach, puncture is initiated using the short-axis view; then, by rotating the ultrasound probe by 90°, the needle is advanced into the target vein using the long-axis view. For ultrasound-guided central venous catheterization in adult patients, the posterior wall penetration rate of this combined approach is reportedly comparable to that of the long-axis in-plane approach and lower than that of the short-axis out-of-plane approach [17, 18]. Furthermore, the first-attempt success rate of the combined approach is reportedly higher than that of the short-axis out-of-plane approach in premature neonates [19]. However, to date, no reported studies have compared the posterior wall penetration rates between these approaches for ultrasound-guided internal jugular vein catheterization in infants and small children.

We hypothesized that the combined approach would not be inferior to the long-axis in-plane approach in preventing posterior wall penetration of the internal jugular vein in infants and small children. The aim of this randomized controlled trial was to compare the posterior wall penetration rates between the long-axis in-plane and combined approaches in infants and small children.

## Materials and methods

### Ethical considerations

This study was performed in accordance with the principles of the Declaration of Helsinki and conducted with approval from the Institutional Review Board of Osaka Women’s and Children’s Hospital, 840 Murodo-cho, Izumi, Osaka 594–1101, Japan (Institutional Review Board #1280/2020, date of approval: January 31, 2020). Written informed consent was obtained from the parents of each patient participating in the trial. This trial was registered before patient enrollment in the University Hospital Medical Information Network Clinical Trials Registry (UMIN000039387; Principal investigator: Jun Takeshita; date of registration: February 4, 2020; https://upload.umin.ac.jp/cgi-bin/ctr/ctr_view_reg.cgi?recptno=R000044907). This manuscript adheres to the Enhancing the Quality and Transparency of Health Research guidelines.

### Trial design and participants

**Fig 1:**
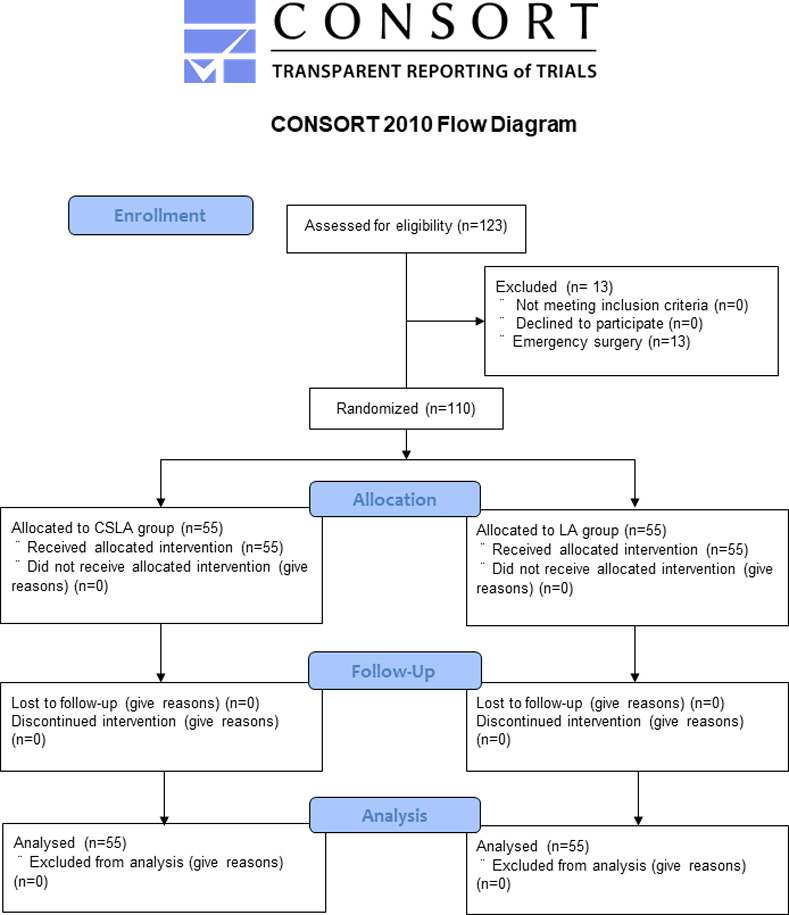
Flowchart of the randomized controlled trial. CSLA, combined short-axis out-of-plane and long-axis in-plane approach; LA, long-axis in-plane approach.

This randomized controlled trial was performed in the operating room of Osaka Women’s and Children’s Hospital from February 2020 to January 2021. Pediatric patients aged <5 years who underwent cardiovascular surgeries and required central venous catheter insertion were included. Emergency surgery was considered as an exclusion criterion (Fig 1).

### Randomization and blinding

In total, 110 eligible patients were randomly allocated to the combined short-axis out-of-plane and long-axis in-plane group (CSLA group) or the long-axis in-plane approach group (LA group). An anesthesiologist who did not participate in the puncturing procedures used computer-generated permuted blocks without stratification to allocate the patients. The allocation data were concealed in sequentially numbered opaque envelopes and opened immediately after general anesthesia induction.

### Interventions

Puncturing procedures were performed by six anesthesiologists with 2–4 years of experience as pediatric anesthesiologists. Each of them had performed >20 ultrasound-guided central venous catheterizations using the long-axis in-plane and short-axis out-of-plane approaches. They had no experience with the combined short-axis out-of-plane and long-axis in-plane approach. Before initiating the trial, the anesthesiologists practiced the combined short-axis out-of-plane and long-axis in-plane approach with a simulator (AGL800 UGP-GEL; ALFABIO Co., Ltd., Maebashi, Japan).

After general anesthesia induction and tracheal intubation, the patient’s head was rotated approximately 45° to the contralateral side and fixed with tape, and a small, rolled towel was placed under the patient’s neck. After achieving the Trendelenburg position (15–20°) and sterilization with 1% chlorhexidine-83% ethyl alcohol, the procedures were initiated. The Sonosite M-Turbo Ultrasound System with an SLAx/13-6 MHz hockey-stick type transducer (38×12.5 mm) (FUJIFILM Medical Co., Ltd., Tokyo, Japan), a 22-gauge needle (Jelco Plus; Smiths Medical Japan, Tokyo, Japan), and a central venous catheter (SMAC Plus; Nippon Covidien Ltd., Tokyo, Japan) were used for ultrasound-guided puncturing procedures.

In the LA group, the ultrasound-guided long-axis in-plane approach was used for puncture, as described in our previous report [10]. The operator inserted the puncture needle at approximately 30–45° and advanced it until it penetrated the anterior wall of the vein while visualizing the long-axis view of the entire needle and the vein [21]. After confirming the blood backflow through the connected syringe, the operator advanced the needle a little further to insert the outer catheter sufficiently into the vein under ultrasound guidance. Subsequently, the operator removed the inner stylet, confirmed blood backflow, and inserted a guidewire into the vein. In the absence of blood backflow, the operator withdrew the catheter while aspirating the blood with a syringe. After the blood was aspirated via the syringe, the guidewire was inserted. In such cases, we defined that posterior wall penetration had occurred, as described in previous studies [10, 17, 18, 20]. If blood return was not confirmed despite withdrawing the catheter to the skin surface, we considered that this attempt had failed, and the next puncture attempt was initiated. Successful guidewire insertion was validated using the long-axis view [21]. In cases where the guidewire could not be inserted into the vein within 20 min, we considered that this attempt had failed.

**Fig 2:**
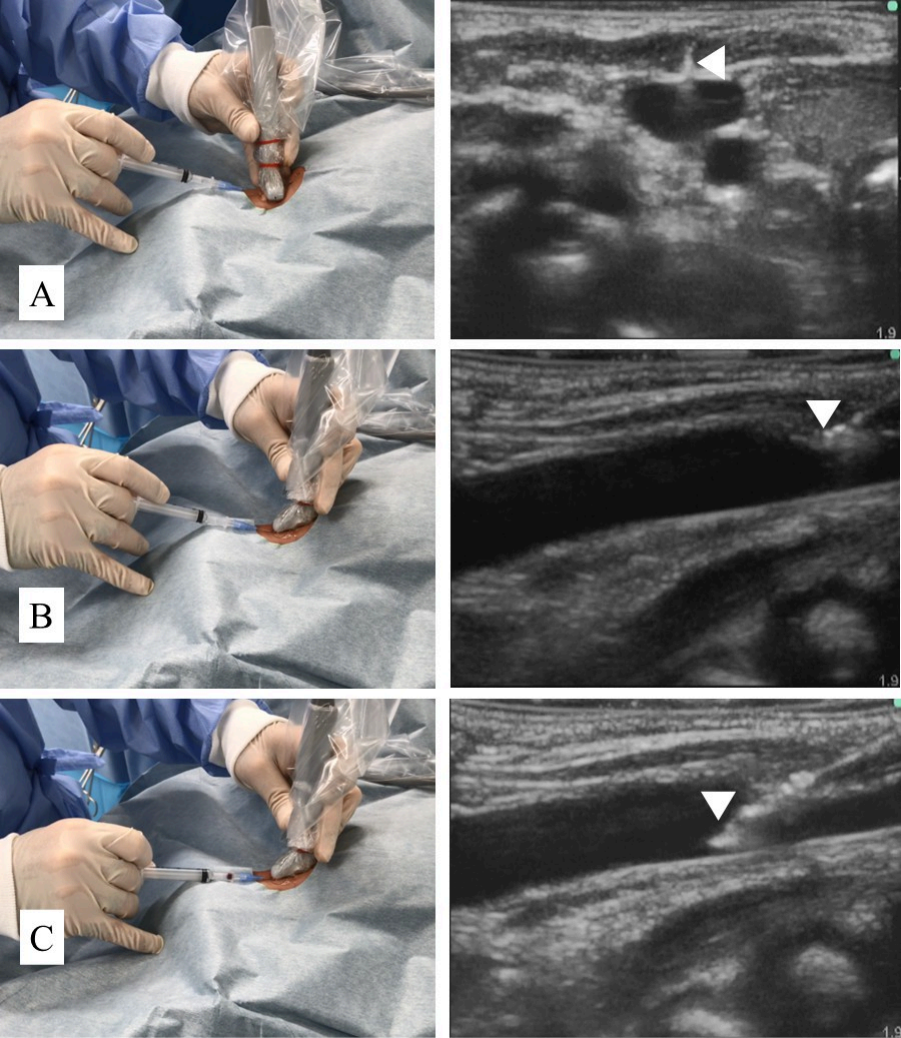
Combined short-axis out-of-plane and long-axis in-plane approach. (A) The needle tip (arrowhead) is visualized as a white dot on the midline axis of the vein in the short-axis view. (B) After rotating the transducer by 90°, the entire length of the needle, including the needle tip (arrowhead) and the vein, are visualized in the long-axis view. (C) The needle tip (arrowhead) is visualized penetrating the anterior wall of the vein.

In the CSLA group, puncture was performed using the ultrasound-guided combined short-axis out-of-plane and long-axis in-plane approach, which has been described previously [17, 18]. After visualizing the short-axis view of the internal jugular vein, the operator inserted the puncture needle at approximately 30–45°. Next, after visualizing the needle tip between the skin surface and the anterior wall of the vein in the short-axis view (Fig 2A), the operator rotated the transducer by 90° and visualized the long-axis view of the entire needle and the vein (Fig 2B). Following this, the operator advanced the needle until it penetrated the anterior wall of the vein (Fig 2C). The subsequent process and the definition of posterior penetration were the same as those for the long-axis in-plane approach.

**Fig 3:**
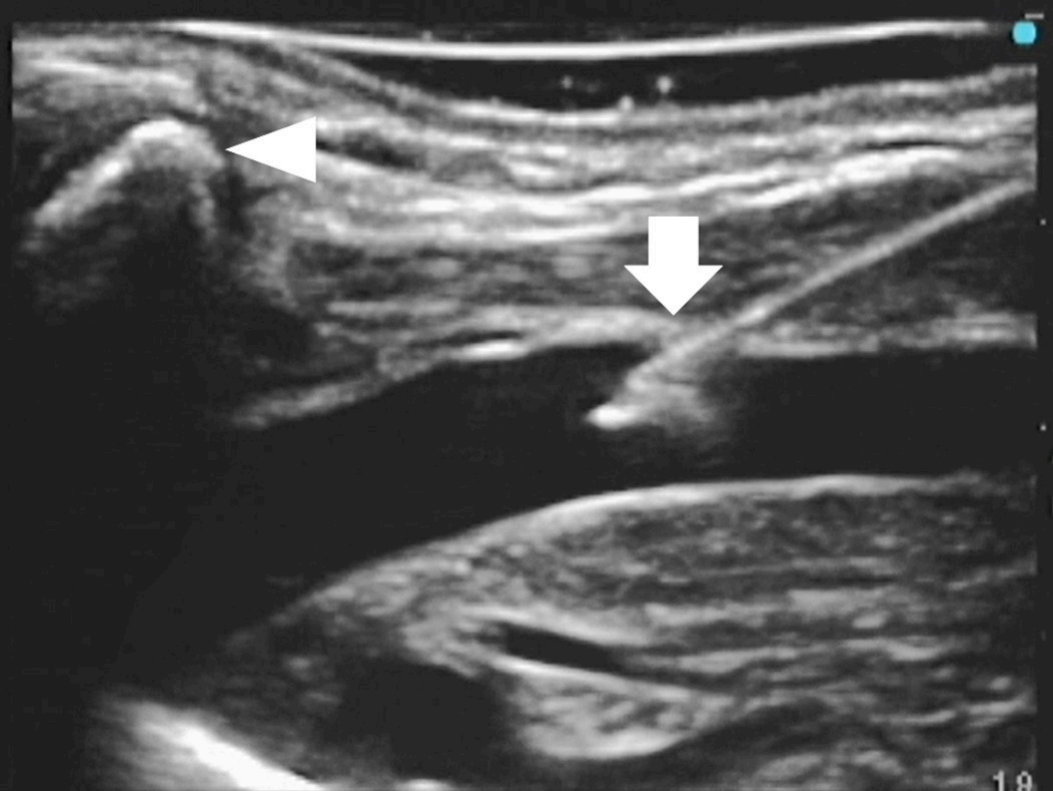
The long-axis view of the internal jugular vein and the needle. The transducer is positioned over the clavicle (arrowhead), and the internal jugular vein and the needle (arrow) are visualized clearly.

For each group, if the puncture space was deemed too narrow to handle the needle and the transducer, the operator positioned the transducer more caudally, over the clavicle (Fig 3) [10]. If the entire needle and the vein were not displayed simultaneously on the ultrasound screen before penetrating the vein, the operator withdrew the needle up to the skin surface. We considered that this attempt had failed, and the next puncture attempt was initiated.

### Measurements

We recorded posterior wall penetration, first-attempt and overall success within 20 min, the number of attempts, scanning duration (from the beginning of placing the ultrasound transducer to the beginning of needle insertion into the skin), puncture duration (from the beginning of needle insertion into the skin to the guidewire confirmation using ultrasonography), and total procedure duration (scanning duration plus puncture duration). Additionally, the perpendicular venous diameter, transverse venous diameter, and subcutaneous venous depth using the short-axis view before skin sterilization were measured by an assessor who did not participate in the punctures.

### Outcomes

The primary outcome was the posterior wall penetration rate in the non-inferiority test. We defined posterior wall penetration above in the Interventions subsection. The secondary outcomes included the first-attempt and overall success (within 20 min) rates, number of attempts, scanning duration, puncture duration, and total procedure duration. We recorded all ultrasound images and puncturing procedures on video. An assessor who did not participate in the punctures assessed the outcomes.

### Sample size

In our previous study, the long-axis in-plane approach resulted in approximately 30% reduction of the posterior wall penetration rate compared to the corresponding after performing the short-axis out-of-plane approach in infants and small children [10]. We speculated that the effect of the combined approach on reduction of posterior wall penetration is more than half of the long-axis in-plane approach (15% reduction). We assumed that the posterior wall penetration rates were 10%, 25%, and 40% in the long-axis in-plane, combined, and short-axis out-of-plane approaches, respectively. Therefore, we set the non-inferiority margin as 15%. Based on this assumption, the estimated number of patients per group was 50 to provide 80% power at an α-level of 0.05. Allowing for potential dropouts, we enrolled 110 patients. The sample size calculation was performed using StatFlex version 6.0 (Artech Co., Ltd., Osaka, Japan).

### Statistical analysis

The posterior wall penetration rate and first-attempt and overall success (within 20 min) rates were compared using Fisher’s exact test or the chi-square test. The number of attempts, scanning duration, puncture duration, and total procedure duration were compared using the Mann‒Whitney *U* test. Statistical analyses were performed using StatFlex version 6.0 (Artech Co., Ltd.). Values are expressed as medians [interquartile ranges] or numbers (proportions). A *P*-value <0.05 was considered indicative of statistical significance.

## Results

The 110 included patients were randomized to the CSLA (*n* = 55) or LA (*n* = 55) group from February 2020 to January 2021 (Fig 1). The patients’ demographic and clinical characteristics are presented in Table 1. There were no significant differences in the characteristics between the two groups.

**Table 1:** Patients’ demographic and clinical characteristics.

Parameters	CSLA group (*n* = 55)	LA group (*n* = 55)	*P*-value
**Sex (male)**	38 (69.1%)	38 (69.1%)	1
**Age (months)**	6 [3–14]	5 [1.3–14]	0.9
**Height (cm)**	61.5 [54–73.4]	62.6 [52.7–72.9]	0.87
**Weight (kg)**	5.4 [4.0–8.6]	5.5 [3.7–8.4]	0.68
**Weight <5 kg**	25 (45.5%)	21 (38.2%)	0.44
**RACHS-1 category (1/2/3/4/5/6)**	5/20/22/5/0/3	8/20/23/2/0/2	0.82
**Previous central venous catheterization**	10 (18.2%)	11 (20%)	0.81
**Side (right)**	38 (69.1%)	37 (67.3%)	0.84
**Subcutaneous venous depth (mm)**	5.3 [4.9–6.2]	5.6 [5.2–6.7]	0.1
**Perpendicular venous diameter (mm)**	4.4 [3.8–5.2]	4.5 [3.9–5.3]	0.37
**Transverse venous diameter (mm)**	5.9 [4.9–7.4]	6.3 [5.1–7.0]	0.6

Data are expressed as medians [interquartile ranges] or numbers (proportions).

CSLA, combined short-axis out-of-plane and long-axis in-plane approach; LA, long-axis in-plane approach; RACHS-1, Risk Adjustment for Congenital Heart Surgery-1.

The posterior wall penetration, first-attempt success, overall success rates within 20 min, and number of attempts did not differ significantly between the CSLA and LA groups. The scanning duration was significantly longer in the LA than in the CSLA group. The puncture duration was significantly longer in the CSLA group than in the LA group. As a result, the total procedure duration was not significantly different between the CSLA and LA groups (Table 2). No adverse events, such as pneumothorax, common carotid artery puncture, and vertebral artery puncture, occurred during this study.

**Table 2:** Comparison of the CSLA and LA approaches.

Parameters	CSLA group (*n* = 55)	LA group (*n* = 55)	*P*-value
**Posterior wall penetration**	3 (5.5%) 95% CI: 1.1–15.1%	2 (3.6%) 95% CI: 0.4–12.5%	0.65
**First-attempt success**	52 (94.5%) 95% CI: 84.9–98.9%	51 (92.7%) 95% CI: 82.4–98.0%	0.70
**Overall success within 20 min**	55 (100%) 95% CI: 93.5–100.0%	54 (98.2%) 95% CI: 90.3–100.0%	0.32
**Number of attempts**	1 [1–1, 1–3]	1 [1–1, 1–3]	0.72
**Scanning duration (min)**	21 [16.5–34.8]	47 [29.3–65.0]	<0.0001
**Puncture duration (min)**	114 [83–170]	74 [52.3–117.3]	0.0002
**Total procedure duration (min)**	141 [99.0–197.8]	118 [88.5–195.5]	0.14

Data are expressed as medians [interquartile ranges, ranges], medians [interquartile ranges], or numbers (proportions).

CI, confidence interval; CSLA, combined short-axis out-of-plane and long-axis in-plane approach; LA, long-axis in-plane approach.

## Discussion

This study demonstrated that the ultrasound-guided combined short-axis out-of-plane and long-axis in-plane approach resulted in a low posterior wall penetration rate, similar to that of the long-axis in-plane approach, for internal jugular vein catheterization in infants and small children. No significant differences were observed in the first-attempt and overall success (within 20 min) rates and the number of attempts between the two groups. The scanning duration was significantly shorter when using the combined short-axis out-of-plane and long-axis in-plane approach. The puncture duration was significantly shorter in the long-axis in-plane approach. Consequently, the procedure duration, which is the sum of these two durations, was not significantly different between the two approaches.

The advantage of the ultrasound-guided long-axis in-plane approach for central venous catheterization in infants and small children is that the real needle tip can be visualized relatively easily, which leads to the prevention of posterior wall penetration [10]. The operator must precisely align the three axes (i.e., the target vein, the transducer, and the needle), which may be challenging in pediatric patients, owing to the small venous diameter [16]. In the combined approach, the longitudinal plane of the needle and the vein can be aligned using the short-axis view, and by rotating the probe, the ultrasound beam can be aligned to those two axes. Therefore, using this approach, the three axes can be relatively easily aligned even by a novice operator. This may contribute to accurate and easy visualization of the long-axis view of the vein and needle, thus, preventing posterior wall penetration. One recent study also showed that the combined short-axis out-of-plane and long-axis in-plane approach had a higher first-attempt success rate than that of the short-axis out-of-plane approach (71.1% vs. 46.7%) for ultrasound-guided central venous catheterization in premature neonates [19]. Thus, the combined short-axis out-of-plane and long-axis in-plane approach might be feasible and useful, even for patients younger than those in our study population. Although the rate of complications owing to posterior wall penetration, such as pneumothorax, common carotid artery puncture, and vertebral artery puncture, is extremely low [12, 13], these complications may result in serious consequences. Operators should prevent posterior wall penetration by carefully visualizing the needle tip in the long-axis view using either approach to avoid these complications.

Herein, the first-attempt and overall success (within 20 min) rates and the number of attempts were not significantly different between the two approaches. Puncture durations were significantly shorter in the long-axis in-plane approach; however, procedure durations were not significantly different between the two approaches, owing to the shorter scanning duration in the combined short-axis out-of-plane and long-axis in-plane approach. The long-axis in-plane approach required a longer scanning duration, owing to difficulties in aligning the midline of the longitudinal plane of the target vein and ultrasound transducer. Furthermore, operators without prior experience in the combined short-axis out-of-plane and long-axis in-plane approach can easily learn this approach after a short introduction. Hence, the combined short-axis out-of-plane and long-axis in-plane approach can serve as an alternative approach for central venous catheterization in pediatric patients.

This study had one main limitation. We defined posterior wall penetration as described in previous studies [10, 17, 18, 20]. However, the exact rate cannot be determined without actually observing the vascular lumen, and our definition of posterior wall penetration might have resulted in its underestimation.

In conclusion, the ultrasound-guided combined short-axis out-of-plane and long-axis in-plane approach can be as effective in preventing posterior wall penetration of the internal jugular vein as the long-axis in-plane approach; thus, it can serve as an alternative approach for ultrasound-guided central venous catheterization in infants and small children.

## Supporting information

S1 File (DOCX)

S2 File (DOC)

S1 Checklist CONSORT 2010 checklist of information to include when reporting a randomised trial*. (DOC)
