# Supplementary material for: Combined short-axis out-of-plane and long-axis in-plane approach versus long-axis in-plane approach for ultrasound-guided central venous catheterization in infants and small children: A randomized controlled trial
Source: PLoS One. 2022 Sep 30;17(9):e0275453. doi: 10.1371/journal.pone.0275453 (PMC9524688; doi:10.1371/journal.pone.0275453)
Supplement: S1 File — (DOCX) [file pone.0275453.s001.docx]

To Institutional Review Board of Osaka Women’s and Children’s Hospital

Department of Anesthesiology

Jun Takeshita

The name of study:

Combined short-axis out-of-plane and long-axis in-plane approach versus long-axis in-plane approach for ultrasound-guided central venous catheterization in infants and small children: a randomized controlled trial

Background:

Central venous catheterization in pediatric patients is an essential technique for perioperative and intensive care management, but is more difficult than in adult patients because of the smaller vessel diameter. The use of an ultrasound-guided technique has been reported to increase the success rate compared to the landmark technique. There are two types of ultrasound-guided puncture: the short-axis out-of-plane approach and the long-axis in-plane approach. In the short-axis out-of-plane approach, the positional relationship between the target vessel and surrounding structures is easy to understand, and it is easy to align the center of the vessel with the direction of needle. However, it is difficult to visualize the true needle tip, and the rate of unintentional penetration of the posterior wall of the vessel is high, which may cause complications. In the long-axis in-plane, the entire needle including the needle tip and the long-axis image of the target vessel are always depicted from the moment of puncture, which makes it easy to identify the needle tip position and prevents posterior wall penetration, but it is technically difficult. We previously devised an ultrasound-guided central venous catheterization in adults using a combination of the short-axis out-of-plane and long-axis in-plane approaches, which we named the combined approach, and reported that it reduced the rate of posterior wall penetration compared with the short-axis out-of-plane approach.

The use of the combined approach for ultrasound-guided central venous catheterization in pediatric patients is expected to decrease the rate of the posterior wall penetration, similar to the long-axis in-plane approach.

Objective:

To determine whether the use of the combined approach for ultrasound-guided central venous catheterization in pediatric patients reduces the rate of posterior wall penetration as well as the long-axis in-plane approach.

Patients:

110 pediatric patients aged <5 years who underwent cardiovascular surgeries and required central venous catheter insertion were included.

Method:

After induction of general anesthesia, ultrasound-guided central venous catheterization is performed. The subjects will be divided into two groups: LA group (long-axis in-plane approach) and CSLA group (combined approach). If the guidewire is not successfully inserted within 20 minutes of the start of ultrasound angiography, the placement will be considered a failure and the study will be terminated. The two groups will be compared for posterior wall penetration rates, first attempt and overall success rates, puncture time, and number of attempts.

The design of the study:

Randomized controlled study

Study endpoint

The primary outcome was the posterior wall penetration rate. The secondary outcomes included the first-attempt and overall success (within 20 min) rates, number of attempts, scanning duration, puncture duration, and total procedure duration.

Study period

After approval for research implementation - March 31, 2023
