# Supplementary material for: Combined short-axis out-of-plane and long-axis in-plane approach versus long-axis in-plane approach for ultrasound-guided central venous catheterization in infants and small children: A randomized controlled trial
Source: PLoS One. 2022 Sep 30;17(9):e0275453. doi: 10.1371/journal.pone.0275453 (PMC9524688; doi:10.1371/journal.pone.0275453)
Supplement: S2 File — (DOC) [file pone.0275453.s002.doc]

（様式１）　　　　　　　　　　　　　　　　　　　　　　　　　　　　　　　　　ver9.0

地方独立行政法人大阪府立病院機構大阪母子医療センター倫理審査申請

地方独立行政法人大阪府立病院機構大阪母子医療センター総長　様

　　　　　　　　　　　　　　　　　　　　　　　　申請者　　　　　竹下　淳

　　　　　　　　　　　　　　　　　　　　　　　　　所　属　麻酔科

　　　　　　　　　　　　　　　　　　　　　　　　　職　名　医長

　　　　　　　　　　　　　　　　　　　　　　　　　メールアドレス　t-k-s-t@koto.kpu-m.ac.jp

　　　　　　　　　　　　　　　　　　　　　　　　　研究倫理セミナー受講番号 第18002号

　地方独立行政法人大阪府立病院機構大阪母子医療センター倫理委員会規程による審査を申請します。

| １．研究，医療行為等課題名　小児患者の超音波ガイド下中心静脈カテーテル留置：交差平行法と平行法の比較（ランダム化比較試験） | ※受付番号 |
| --- | --- |
| ２．公開用課題名　小児患者の超音波ガイド下中心静脈カテーテル留置：交差平行法と平行法の比較（ランダム化比較試験） | |
| ３．研究代表者名　竹下　淳　所属　麻酔科　職名　医長　役割　総括、解析、データ収集、IC取得 | |
| ４．研究分担者名　橘　一也　所属　麻酔科　　職名　主任部長　　役割　データ収集、解析、助言  （3.4　研究責任者(研究の実施に携わり当センターで研究業務を統括する者)の名前の前に○を付けてください） | |
| ５．研究等の種類   - - ☑人を対象とする医学系研究   ☑新たに試料・情報を取得して行う研究  →対象者のリスク・負担  （介入：☑あり □なし　侵襲：□あり ☑軽微 □なし　人体取得試料：□あり ☑なし）  □既存試料・情報を用いる研究  →既存試料・情報　　　（□他機関へ提供　□他機関から取得　　□当センターで利用）   - - ヒトゲノム・遺伝子解析研究   □当センターで遺伝子解析実施　□試料・情報を他機関へ提供　□試料・情報を他機関から取得   - - その他（医療行為等）   　添付書類  　　人を対象とする医学系研究に関する倫理指針による審査対象研究  ☑計画書 ☑説明文書 ☑同意文書 ☑同意撤回文書 □症例調査用紙  □情報公開資料（□様式8.1 □様式8.2 □その他）□試料・情報の提供記録(様式11等)  □モニタリング手順書(計画書) □監査手順書(計画書)  ヒトゲノム・遺伝子解析研究に関する倫理指針による審査対象研究  □ヒトゲノム・遺伝子解析研究計画書 □説明文書　□同意文書　□同意撤回文書　□情報公開資料  医療行為  　　　□計画書　　□説明文書　□同意文書　□同意撤回文書　□症例要約    その他（例：共同研究機関の研究実施許可書、共同研究機関作成の計画書、アンケート調査票、参考文献など）  　　　□（　 　　　　　　　　　　　　　　　　　　　　　　　　　　　） | |
| ☑．本センターのみの人を対象とする医学系研究  □．他の研究機関と共同して実施される研究で、共同研究機関で実施の承認を受けていないもの  □．他の研究機関と共同して実施される研究で、既に共同研究機関で実施の承認を受けているもの  □．厚生労働省・文部科学省等の公的班研究  　□．その他（含．個別症例に対する新医療等の医療行為） | |

| 課題の概要（1,000字程度）  ＜背景＞  小児患者の中心静脈カテーテル留置は周術期及び集中治療管理に必須の手技であるが、血管径が細いため成人患者に比べて難易度が高い。超音波ガイド下法を用いることにより、ランドマーク法に比べて成功率が上昇することが報告されている。超音波ガイド下穿刺には交差法と平行法がある。交差法では標的血管と周囲の構造物との位置関係がわかりやすく、血管の中心と針の進行方向を合わせるのが容易であるが、真の針先の描出が難しく、意図せずして血管後壁を貫通してしまう率は高く、合併症を起こす可能性がある。平行法では、穿刺の瞬間から、針先を含めた針全体と標的血管の長軸像が常に描出されるため、針先端位置がわかりやすく、血管後壁貫通を防ぐことができるが、技術的に難しい。我々は以前、成人の超音波ガイド下内頸静脈穿刺において、交差法と平行法を組み合わせた穿刺法を考案して交差平行法と命名し、交差法と比べて血管後壁貫通率が減少することを報告している。具体的には、交差法で穿刺を開始して針先が血管前壁に向かっていることを確認した後に超音波プローブを90°回転させて平行法に移行し、針全体を長軸像で描出しながら進めていく穿刺法である。平行法単独では超音波断面、血管軸、穿刺針と３つの軸を一致させる必要があるが、初めに交差法で血管軸と穿刺針の軸を合わせておくことにより、穿刺が容易になると考えられる。  小児患者の超音波ガイド下内頸静脈穿刺に交差平行法を用いることにより、平行法と同様に血管後壁貫通率が減少することが予想される。  　＜研究内容＞  目的：小児患者の超音波ガイド下内頸静脈穿刺に交差平行法を用いることにより、平行法と同様に血管後壁貫通率が減少するかどうかを検討する。  対象者：大阪母子医療センター手術室で外科手術を受け、麻酔導入後に内頸静脈より中心静脈カテーテルを留置される5歳未満の患者。  対象者数　(多施設共同研究の場合は、母子医療センターと全体の対象者数)：110名  方法：対象を2群にランダムに分ける。  全身麻酔導入後、超音波ガイド下に内頸静脈穿刺を施行する。対象をL群(平行法を用いて穿刺する)とC群(交差平行法を用いて穿刺する)の2群に分ける。ランダム化は、穿刺施行者とは異なる研究分担者がブロックランダム化にて行う。まず、コンピューターを用いて、2個ずつのブロックを55個あらかじめ作成する。そしてそれに基づいて、L群かC群かに分ける。ランダム化の結果は中の見えない封筒に入れ、麻酔導入直前に開封し、穿刺施行者は穿刺直前に対象者の穿刺方法を知らされる（盲検化）。穿刺施行者は複数名で、一人の患者につき一人の施行者が行う。超音波による血管描出開始から20分以内にガイドワイヤー挿入に成功しなければ、留置は失敗とし、そこで研究は終了とする。その後は研究とは無関係に、同じ部位または別の部位で、指導医が超音波ガイド下に、手術中や手術後の管理のために必要な中心静脈カテーテルを留置する。血管後壁貫通率、初回留置成功率、最終留置成功率、穿刺時間、穿刺回数について両群で比較する。  研究期間：研究実施許可後～2023年3月31日  研究デザイン：ランダム化比較試験  評価項目：血管後壁貫通率、初回留置成功率、最終留置成功率、穿刺時間、穿刺回数  対象者への説明・同意方法：文書を用いて説明し、同意書を取得  ＜対象者の安全性・不利益に対する配慮＞  小児の超音波ガイド下内頸静脈穿刺による中心静脈カテーテル挿入は通常の診療の範囲を超えず、超音波を用いることの合併症や不利益はなく、触知下穿刺に比べて成功率が高いことが知られている。どちらの群に割り付けられても触知下穿刺よりは安全性が高く、対象者の安全性や不利益に対する特段の配慮は必要ないと考えられる。  ＜研究中及び研究後の試料・情報の保管・廃棄（多施設共同研究の場合は他施設の状況も含む）＞  ネットワークから切り離されたコンピューターを使用して記録された外部記憶媒体（USBなど）を施錠可能な場所に厳重に保管する。研究成果発表後、10年保管し、その後、適切に廃棄する。  ＜研究で扱う個人情報等＞  氏名、ID、年齢、性別、身長、体重、血管径、深さ、疾患名  ＜個人情報等の適正な取り扱い、保管、廃棄＞漏洩、盗難に注意し、ネットワークから切り離されたコンピューターを使用して記録された外部記憶媒体（USBなど）を施錠可能な場所に厳重に保管する。研究成果発表後、10年保管し、その後、適切に廃棄する。 |
| --- |
